# The Effect of Visual Feedback on Writing Size in Parkinson's Disease

**DOI:** 10.1155/2015/857041

**Published:** 2015-06-17

**Authors:** Adriaan R. E. Potgieser, Elizabeth Roosma, Martijn Beudel, Bauke M. de Jong

**Affiliations:** Department of Neurology, University Medical Center Groningen, University of Groningen, Hanzeplein 1, 9700 RB Groningen, Netherlands

## Abstract

Parkinson's disease (PD) leads to impairment in multiple cognitive domains. Micrographia is a relatively early PD sign of visuomotor dysfunction, characterized by a global reduction in writing size and a decrement in size during writing. Here we aimed to investigate the effect of withdrawal of visual feedback on writing size in patients with PD. Twenty-five patients with non-tremor-dominant PD without cognitive dysfunction and twenty-five age-matched controls had to write a standard sentence with and without visual feedback. We assessed the effect of withdrawal of visual feedback by measuring vertical word size (i), horizontal length of the sentence (ii), and the summed horizontal word length without interspacing (iii), comparing patients with controls. In both patients and controls, writing was significantly larger without visual feedback. This enlargement did not significantly differ between the groups. Smaller handwriting significantly correlated with increased disease severity. Contrary to previous observations that withdrawal of visual feedback caused increased writing size in specifically PD, we did not find differences between patients and controls. Both groups wrote larger without visual feedback, which adds insight in general neuronal mechanisms underlying the balance between feed-forward and feedback in visuomotor control, mechanisms that also hold for grasping movements.

## 1. Introduction

It is well-recognized that Parkinson's disease (PD) is not only characterized by motor symptoms but inflicts impairment in multiple other domains. Cognitive dysfunction such as visuospatial impairment is evident, also in patients without signs of dementia [1]. Even in mild PD, disturbed visuospatial perception can be observed [2]. Micrographia is a common sign in early PD, characterized by a global reduction in writing size and/or a decrement in size during writing [3]. Aside from such impairments, PD patients have different responses to external visual stimuli compared to control subjects [4], pointing towards altered visuomotor integration in PD [5]. A characteristic example of this is kinesia paradoxa; a strong improvement of motor symptoms after sensory stimuli. Given this disturbed visuomotor integration, micrographia may not merely point at motor dysfunction but reflects a consequence of higher order visuospatial dysfunction. This concept is supported by the temporary increase of a patient's size of writing when asked to do so [6].

In this concise experiment, we aimed to establish the effect of visual feedback on the size of writing in PD patients compared to age-matched control subjects. In line with a previous experiment, we expected patients with PD to exhibit larger writing without visual feedback, while such effect is less evident in control subjects [7].

## 2. Methods

Twenty-five patients with PD (mean age 64.4 years, SD 8.7) and twenty-five age-matched controls (mean age 64.0 years, SD 9.1) were included in this study. The scales for outcomes in Parkinson's disease, cognition (SCOPA-Cog, range 0–43), were used to compare cognition in PD patients with controls. Patients were recruited from the Movement Disorders Unit of the Neurology Department of the University Medical Center Groningen (UMCG). From this sample, both tremor-dominant PD without bradykinesia and patients receiving deep brain stimulation were excluded. Severity of motor symptoms was assessed using part III of the Movement Disorder Society Unified Parkinson's Disease Rating Scale (MDS-UPDRS, range 0–132). Dose intensity of different dopaminergic drug regimens of patients was expressed in a total levodopa equivalent dose [8]. None of the participants had other neurological, psychiatric, ophthalmological, or musculoskeletal comorbidity that interfered with the task. Handedness was assessed using the Edinburgh Handedness Inventory [9]. Three patients and two controls were left-handed. Patients were tested approximately two hours before their end of dose of dopaminergic medication.

Subjects had to write the sentence “Het is mooi weer vandaag” (Dutch for “it is nice weather today”) with their dominant hand on a blank paper without parallel lines, successively both with and without visual feedback. The acquisition was done by three investigators (Elizabeth Roosma, Adriaan R. E. Potgieser, and Bauke M. de Jong). The absence of visual feedback was achieved by keeping a blank paper above the writing hand. Paper without parallel lining was chosen because patients may increase the amplitude of writing when requested to write between lines.

The experiment was approved by the local medical ethical committee of the UMCG. All patients and ten healthy subjects that were tested with the SCOPA-Cog gave written informed consent according to the declaration of Helsinki. Fifteen control subjects were recruited in addition to the original protocol without, however, SCOPA-Cog assessment. This implied that we refrained from a formal written informed consent, in accordance with Dutch legislation, because no person-specific details other than age, sex, and handedness were filed.

### 2.1. Data Acquisition and Statistical Analysis

Data were tested for normality with the Kolmogorov-Smirnov test. Age and SCOPA-Cog were normally distributed which allowed testing for differences between groups with independent-samples *t*-tests. The Edinburgh Handedness Inventory was not normally distributed and tested with the Mann-Whitney *U* test. Gender was compared using the Chi-square test.

To assess the size of writing, the length of the sentence and summed horizontal word length without interspacing, as well as height of the letters, were measured. These measurements were made blinded without knowledge concerning the presence or absence of visual feedback and whether the subject was a patient or control subject. Vertical size of writing was assessed using the vowels “i,” “o,” “e,” and “a.” These data were not normally distributed. Differences in size with and without visual feedback for subjects were calculated using the Wilcoxon Signed Ranks test, while statistical testing between patients and controls was done with independent samples Mann-Whitney *U* tests. A ratio of sizes in the absence versus presence of feedback was calculated per subject, enabling the comparison between PD patients and the control group with independent-samples Mann-Whitney *U* tests. Normal writing (with feedback) was correlated with the MDS-UPDRS part III using Spearman's rho. A possible decrement during writing in PD patients was measured with a Wilcoxon Signed Ranks test, comparing the vertical size of the vowels in “mooi” and “vandaag” in the condition with visual feedback. The Statistical Package for the Social Sciences (SPSS; IBM Corp, Armonk, NY) version 22 was used for all analyses. A *p* value <0.05 was considered statistically significant.

## 3. Results

The analysis of subject characteristics showed that the PD and healthy subject groups were matched for gender, age, handedness, and cognitive function assessed by SCOPA-Cog. These and additional characteristics are summarized in Table 1.

### 3.1. Size of Writing

Measurements along the vertical and horizontal axes of writing showed that without visual feedback both control subjects and PD patients wrote significantly larger along both the horizontal and vertical axes (Table 2). The two groups did not differ in the relative increase of writing size when this was performed without visual feedback (Table 2). Although performance with visual feedback suggested a tendency to smaller PD handwriting, the two groups did not significantly differ in writing size. No decrement in vertical size (mean ± SD) was found in writing of the PD patients: 0.31 ± 0.10 cm for the measurement on the sentence onset and 0.31 ± 0.11 at the end of the sentence (*p* = 1.0). On the other hand, vertical size of writing with visual feedback negatively correlated with the MDS-UPDRS part III score (*p* < 0.01), annotating smaller writing in patients with a higher MDS-UPDRS score. This correlation remained subthreshold for the horizontal size of writing (*p* = 0.06).

## 4. Discussion

The main finding of our study was the similar enlargement of writing in PD patients and control subjects when it was performed without visual feedback. With feedback, the only significant indicator of micrographia in PD was a correlation between smaller writing and a higher MDS-UPDRS (part III) score. The comparison of PD with control subjects showed a tendency to smaller writing in patients without statistical significance. Furthermore, no decrement was seen in the size of writing in the PD patients during writing. We did, however, not formally assess the possibility of micrographia gradually evolving over a prolonged time of writing in patients. Our research question and method of analysis was restricted to the size component of writing, while it is known from studies using graphic tablets that other parameters can be affected such as the velocity, fluency, and duration of handwriting [3].

At first sight, our findings seem contradictory to the study of Ondo and Satija [7], to our knowledge the only other study assessing the effect of withdrawal of visual feedback. They described that only patients off medication had a larger handwriting in the absence of visual feedback, while such enlargement was not the case in PD patients on medication and controls [7]. Because our patients were tested two hours before the end of dose interval, one might consider them (partly) comparable with the “on” medication group of Ondo and Satija [7]. Moreover, although they did not report a UPDRS score, their patients may have been more severely affected, inferred from a longer disease duration than our patients (means 8.5 versus 6.0 years). Our results indicate that patients with a higher MDS-UPDRS III have a smaller size of normal writing. The largest increase of writing size observed by Ondo and Satija was in patients with the smallest baseline in their study writing [7]. Because our patients did not have evident micrographia, it might still be possible that an extra increase in size of writing without visual feedback indeed particularly occurs in patients with micrographia. This might imply that micrographia and an extra increase in size of writing without visual feedback have a common cause of underlying neuronal dysfunction concerning scaling of size.

Our findings are, on the other hand, in agreement with the observation that healthy controls also write larger without visual feedback [10]. A very recent review on the impact of PD on writing showed that 42% of the studies did not find a significant difference in size of writing between PD patients and controls, while 50% of studies found no difference in size of writing between on and off treatment in PD patients [3]. This might explain why we also did not find differences between the groups.

We excluded patients with tremor-dominant PD without bradykinesia, while Ondo and Satija [7] did not report a distribution of specific PD subgroups. Theoretically, withdrawal of visual feedback might have a more pronounced effect on the size of writing in tremor-dominant patients. On the other hand, the enlargement of writing due to the absence of visual feedback occurred in both groups, adding ground to the idea that size scaling is a basic parameter in visuomotor control dependent on sensory input. In this respect one should consider the concept that motor preparation is not directly matched to visual input but is mediated by an internal reference system onto which visual input and motor output are tuned during visuomotor control [4]. In visuomotor action, movement preparation includes feedforward processing of the sensory consequences of such action [11], while, during the successive stages of performance, actual sensory feedback enables adjustment of motor planning. Without visual feedback, proprioceptive information as well as feedforward mechanisms may thus become more dominant. It is, in this respect, intriguing to speculate on a similar neuronal scaling process in writing and grasping. In the latter, the initial aperture is larger than the target size while, during reaching, it gradually decreases to match the size of the object [12]. One might thus expect that without updated visual feedback, dominance of the initial movement plan results in a persistently enlarged hand aperture. This has indeed been described [13]. A line of further research may focus on mechanisms of size scaling in specific PD subgroups in order to find an explanation for the existing interstudy variability.

## 5. Conclusion

The main finding of our study was a similar enlargement of writing in PD patients and healthy control subjects when performed without visual feedback. Although deviant from previous observations in PD, our findings added insight in general neuronal mechanisms underlying the balance between feed-forward and feedback in visuomotor control. With feedback, the only significant indicator of micrographia in PD was a correlation between smaller writing and a higher MDS-UPDRS (part III) score.

## Figures and Tables

**Table 1:** Subject characteristics.

	Controls (*n* = 25)	PD (*n* = 25)	Difference (*p* value)
Gender, male	14	13	0.78
Age, years	64.0 (9.1)	64.4 (8.7)	0.86
EHI	77.0 (52.2)	80.2 (39.5)	0.95
SCOPA-Cog	28.5 (4.6)^*∗*^	29.9 (4.9)	0.80
UPDRS III	—	22.0 (13.5)	—
Disease duration (years)	—	6.0 (5.1)	—
LED (mg)	—	764 (425)	—

Data presented as means with standard deviation in parentheses where appropriate.

PD: Parkinson's disease; EHI: Edinburgh Handedness Inventory (range −100–+100); SCOPA-Cog: PD Score on cognition (range 0–43); UPDRS III: Unified Parkinson's Disease Rating Scale, part III, concerning motor symptoms (range 0–76); LED: levodopa equivalent dose.

^*∗*^ *n* = 10 for the SCOPA-Cog in this group.

**Table 2:** Size of writing in the presence and absence of visual feedback.

	Controls	PD patients	Difference (*p* value)
*Vertical writing size *			
+ feedback (cm)	0.33 (0.12)	0.31 (0.11)	0.48
− feedback (cm)	0.38 (0.13)	0.38 (0.13)	0.78
Difference (*p* value)	<0.01	<0.01	
Ratio −/+ feedback (%)	118 (20)	122 (22)	0.32

*Horizontal writing size *			
+ feedback (cm)	12.7 (2.3)	11.5 (2.7)	0.11
− feedback (cm)	14.0 (2.8)	13.2 (2.9)	0.44
Difference (*p* value)	<0.01	<0.01	
Ratio −/+ feedback (%)	111 (18)	117 (19)	0.23

*Summed word length without interspacing *			
+ feedback (cm)	9.3 (1.8)	8.4 (2.0)	0.08
− feedback (cm)	10.2 (2.1)	9.2 (2.1)	0.09
Difference (*p* value)	<0.01	0.02	
Ratio −/+ feedback (%)	110 (15)	110 (16)	0.89

Data presented as means with standard deviation in parentheses where appropriate; PD: Parkinson's disease.
